# Ecological Public Health and Participatory Planning and Assessment Dilemmas: The Case of Water Resources Management

**DOI:** 10.3390/ijerph15081635

**Published:** 2018-08-02

**Authors:** Tom Elijah Volenzo, John Odiyo

**Affiliations:** School of Environmental Sciences, University of Venda, Thohoyandou 0950, South Africa; john.odiyo@univen.ac.za

**Keywords:** community empowerment, ecological public health, externalities, IWRM, participatory planning and assessment, public health risks, resilience, water quality related risks

## Abstract

Water is a key driver for socio-economic development, livelihoods and ecosystem integrity. This is reflected in the emergence of unified paradigms such as Integrated Water Resource Management (IWRM) and the weight accorded to it in the Sustainable Development Goals agenda. This paper interrogated the effectiveness of existing participatory planning and assessment models adapted from IWRM model on water quality and public health at community level. The analysis was built around public health ecology perspective and drew useful lessons from critique of basin wide integrated Modeling approaches and existing community participatory models envisaged under Water Users Associations (WUA) in South Africa. We extended the use of political ecology lenses to ecological public health through use of communication for development approaches, to argue that public health risk reduction and resilience building in community water projects require the use of innovative analytical and conceptual lenses that unbundle cognitive biases and failures, as well as, integrate and transform individual and collective agency. The study concludes that the inherent “passive participation” adapted from IWRM model fail to adequately address water quality and public health dimensions in its pillars. Since water quality has direct bearing on disaster risks in public health, building a coherent mitigatory vision requires the adoption of active participatory assessment and planning models that incorporate livelihoods, agency, social learning dynamics and resilience through recognition of communication for development approaches in community empowerment.

## 1. Introduction

Poor management of water resources causes health, environment and economic losses on a scale that impedes development and frustrates poverty reduction efforts [1]. The observation has informed policy action at local and global scales over the last three decades [2]. For example, the Sustainable Development Goals (SDGs) agenda on water seeks to ensure availability and sustainable management of water, sanitation and maintenance of water-related ecosystems. Sustainable management and development of water resources is fundamental to poverty alleviation and inclusive growth [3]. Furthermore, water resources management underpins and interacts with all the pillars of the green economy, including environmental protection, food and energy. The universality of water in terms of social and environmental externalities is largely responsible for emergence of collaborative frameworks, protocols, treaties and convections [4]. Such mechanisms and models have been cascaded to the local levels in many countries, such as, South Africa.

Water is one the natural resources, the management of which is characterized by uncertainties especially in accounting for externalities and predicting the effect of a particular management strategy. The uncertainties are, in part, a product of the growing emphasis on long-term, multiscale and integrative aspects of resource management among researchers, community of practice and policy makers [5]. An externality is present when the production or consumption activities of one economic agent have direct, non-price-mediated effects on the production or consumption activities of another economic agent [6]. The inherent externalities and the attendant social costs in terms of public health risks and water borne disease burden from biological and chemical pollution of ground and surface water resources could be exacerbated through ineffective participation models in the management of water resources. The institutional framing is critical in the analysis of ecological public health perspectives, as well as, risk management at community level.

Suitable policies and laws, viable political institutions, workable financing arrangements, self-governing and self-supporting local systems, and a variety of other institutional arrangements are integral to mitigation of key challenges and risks in water management [7]. Institutions make the rules, values and practices that guide formal and informal organisations [8]. Institutions structure political, economic and social interactions to create order and reduce uncertainty in exchange processes [8]. Institutions consist of both informal constraints (sanctions, taboos, customs, traditions, and codes of conduct), and formal rules (constitutions, laws, property rights). Together with the standard constraints of economics they define the choice set that determine transaction and production costs, as well as, profitability and feasibility of engaging in an economic activity. Given that institutions provide [8] the incentive structure of an economy, they shape the direction of economic change towards growth, stagnation, or decline.

The Integrated Water Resource Management (IWRM) paradigm informs institutional frameworks on water resources management in many countries of the world [1]. IWRM is defined as a process that promotes the coordinated development and management of water, land, and related resources to maximise the resultant economic and social welfare in an equitable manner without compromising the sustainability of vital ecosystems [2]. The paradigm aims at ensuring efficient, equitable and sustainable development and management of the world’s limited water resources and for coping with conflicting demand [1,7,9]. The paradigm is especially invaluable in addressing intricate policy matrices, water related risks and conflict among policy makers, decision makers and researchers [9,10], hence its dominance as a paradigm for transformation of water management globally.

Integration across broad policy sectors requires integration among water sub-sectors referred to as “purposes” of water management. These are water supply, water quality, environmental water control, irrigation, flood control, navigation, hydropower, and recreation. Integration depends on decision problem and involvement of all stakeholders in planning and management processes [1], as well as, a coordinated framework that considers socioeconomic and institutional dimensions of coupled social ecological system [11]. Participatory assessment and planning, as well as, consideration for livelihood lenses and disaster risk reduction paradigms could be critical in the implementation of IWRM across the broad policy sectors, subsector purposes and adaptation to ecological public health perspectives.

Risk and resilience are related and interrelated analytical lenses in public health [12]. Risk and resilience are critical in water resources management due to existence of many drivers of risks and uncertainties. This is particularly critical in rural-based water supply systems. The role of community agency in resilience building has been explored by different authors. Community resilience refers to the existence, development, and engagement of community resources by community members to thrive in an environment characterised by change, uncertainty, unpredictability, and surprise or social sustainability [13]. Community resilience is critical in facing the multiple challenges, risks and uncertainties in water resource management. Community resilience can be enhanced through community-based disaster risk reduction approaches adaptation initiatives [12].

Transformation or empowerment of any given community is seen as a lasting solution to socio-economic challenges. Transforming communities from passive roles to primary and active agents in change management has been shown to positively impact community resilience [13]. This is especially relevant where change or adversity creates vulnerability and increases disaster risks [12]. Coordinated and culturally competent public and private services and institutions are critical elements in the availability of and access to good and high-quality health [12]. Public and private services and institutions include local government, public health and health care, social services, education, public safety, community groups and coalitions, community-based organizations, faith institutions, businesses, and arts institutions. Community resilience approach or framing provides a good framework for conceptualising public health improvements in multi-institutional environment.

The ability to self-organise and capacity to adapt and learn are critical considerations in resilience [14]. Since cognitive failure is recipe for increased future threats and reduced resilience, there is need to consider the diverse economy and livelihood dimensions of a community’s resilience [14]. Though, it is difficult to define which dimensions (community resources, community networks, institutions and services, people-place connections active agents and learning) are relevant in adapting to future changes [15], consideration for behavioural dynamics in the transformative processes is critical as it is integral to social learning and sustainability agenda [16]. This is partly because perception of current resilience may lead to complacency resulting to inaction or maladaptation to systemic changes brought about by multiple interacting processes that cascade and transcend scale and common property resource systems [15]. Water is among the common property resources. The preceding observation is critical in the improvement of IWRM and integration of ecological public health perspectives into water resources management. This is more so in community micro projects and rural water supply systems, which is the focus of this study.

Though IWRM is presented as a unifying paradigm in water resource management, some scholars conclude that it has limited implementation potential to improve management in micro-scale projects. For example [17], attributes the limited potential of IWRM in micro water projects to significant differences and heterogeneity in culture, social norms, and physical attributes, skewed availability of renewable and non-renewable resources, management capacities and types and effectiveness of institutions. However, such limitations can be corrected by focusing on local context, including perspectives that address conflict and recognise negotiation, as well as, focus on divergent values and interests among the stakeholders [18]. Both observations are significant in the current study. As an integrative approach, IWRM can be improved by focussing on transformative processes and structures of a social system.

Public health ecology has been proposed as an appropriate approach for addressing the multiple transitions that currently affect human health and sustainability [19]. The view resonates with analysis in this study on water related public health risks. Exploring the institutional framework in water resource management, its limitations and its ramifications on public health could improve and provide innovative analytical lenses from which ecological public health perspectives are framed and enhanced. As one of the water scarce countries [20] of South Africa provides an important case study for analysing ecological public health perspectives. In addition, South Africa is one of the countries with high morbidity and mortality from water borne diseases [21], especially among formally disadvantaged areas and rural communities. The undesirable conditions are exacerbated by climate change risks [22].

Ecological Public health thinking places particular emphasis on the interplay of ecosystems health and human health, as well as, consideration for economic factors [19] and suggests a paradigm shift through investment in sustainability for a steady state economy from which equitable conditions for health are expected to accrue [23]. In practice ecological public health supports capacity building, coherence and vision into integrative frameworks that address sustainability and disaster risk reduction, e.g., the development of low carbon economy and massive retrofitting of housing [19]. This requires medium and long-term changes with clear shared understanding among public health actors [19]. However, its weakness lies in its failure to recognise the role of individual effort in resilience building [24]. This study attempts to close this gap by focusing on how individual and collective participation in water resources management can be harnessed for positive public health outcomes.

Uncertainty and unpredictable outcomes are embedded in coupled and co-evolving social-ecological interactions [25,26], yet ecological public health depends on successful implementation coexistence of the natural and social dimensions in society [24]. The implicit systems analysis in ecological public health is important in managing social transitions towards healthy habits [24]. Given that power dynamics are inherent in interactions and formal policy arenas and across multiple scale, there is increased need for use of political ecology lenses which ensures the of inclusion of interests and knowledge of state, non-state actors and the community in participatory and adaptive co-management initiatives [27]. This study argues that some of the system analyses are institutional and policy related. Re-engineering institutional frameworks is one of the potential adaptation ‘softwares’ to mitigating public health risks in uncertain environments.

Historically rooted institutional biases are key elements of community health outcomes and the inherent social costs of illness [12]. Some authors [24,28] have argued that ‘ecological public health’ should be the prominent approach to improving health in the 21st Century. However, this observation is based on findings that largely focus on economic dimensions such as the Gross Domestic Product (GNP) and how this affects human health and welfare. The economic, social and environmental factors in South Africa mirrors this analysis especially in relation to water resources and provide a good case study for researchers, community of practice and policy makers. This review provides useful insights into the effect of institutional frameworks on public health from ecological public health perspectives.

There is strong evidence that health is societally determined [29] and that health underpins economics [24,30]. As a one of the integrative model on health issues, ecological public health paradigm is largely informed by the need for coherent advocacy in articulating and prioritising health issues, as well as, need for enhanced political support and action on health matters [24]. Moreover, the visibility and coherence of any integrative models is critical in determining their utility in a dynamic socio-ecological environment. An attempt is made to the institutional perspectives with a view of improving the visibility, coherence and utility of ecological public health perspectives in science-policy-practice environments that are increasingly relying on integrative models.

Focus on local contexts in terms of capacity, decentralisation/devolution, conflict and negotiation among multiple actors with divergent values captures the need to pursue political ecology in participatory approaches [27]. Drawing upon findings on participatory planning from a Water Users Association (WUA) in a South African municipality and basin wide modelling approaches from other regions, existing weaknesses and limitations were identified to suggest a model that can enhance effective participation. In doing so, the study drew from sustainable livelihood frameworks [31], socioecological systems [26] and communication for development paradigms [32,33] to address the limitations of IWRM in relation to ecological health perspectives. The study in particular examined the role of communication for development approaches and the concept of participation as a unifying concept that underpins ecological public health and IWRM. 

The main contribution through the review is in resolving existing policy—practice gaps on integrative models and participatory planning [18]. Focusing on the institutional framework from a participatory angle broadens and enhances the robustness of ecological public health perspective as an integrative model that encapsulates individual knowledge and interests. We extend the concept of political ecology [27], to suggest a conceptual framework that utilises communication for development in unbundling participation dilemmas. To explain participation, we draw on the concept of community empowerment. Following the empowerment approach, we posit that effective participation is critical in unbundling cognitive failure and biases among planners, community, development practitioners, policy and decision makers.

The paper is organized as follows, in the next two sections we review literature related to integration elements and polycentric governance in water resources management to show the need for transformation if water related public health risks are to address at community level. In the third section we outline ecological public health and Participatory Assessment and Planning Dilemmas. In the fourth section we present IWRM in the Context of South Africa’s Community Participation in Development and Management of Water Resources to illustrate the need for innovative approaches in addressing water quality management at community level. In section five, we present case studies of community participation in Limpopo province, South Africa to unbundle participatory dilemmas. We finally present a community-based risk reduction model that interfaces planning and public health in water projects. The central contribution of this review is thus a robust analytical framework for the integration of knowledge, attitudes and behaviour into ecological public health perspective in planning mitigation of water related public health risks.

## 2. Contextualising Critical Issues

### 2.1. Elements of Integration in Water Resources Management

The 1992 United Nations Conference on Environment and Development positioned sustainable livelihoods as a means of linking socioeconomic and environmental concerns [34]. This is relevant to IWRM as it is built around the economic, environmental and social pillars. However, balancing between the three pillars remains a challenge in many countries [1]. This is especially true because IWRM is premised on co-management, adaptive management and collective action approaches. IWRM is consistent with comprehensive or holistic frameworks that recognise cooperation and coordination, decentralisation and grass-roots participation, partnering, public or private enterprise, and watershed focus for planning and management as integrative concepts alongside capacity-building [1].

Adaptive governance focuses on experimentation and learning, by bringing institutions and organizations for collaboration, collective action, and conflict resolution in relation to natural resource and ecosystem management [25]. Adaptation to global environmental change, such as climate change, may include ‘hard approaches’, ‘soft approaches’, and ‘ecosystem-based adaptation’. Hard approaches employ infrastructure or technology in an effort to limit the damages caused by natural disasters. Examples include physical structures such as sea walls and embankments as well as activities such as channel dredging. Soft approaches are behavioural, focusing on limiting exposure through early warning systems, education, and effective planning. In contrast, ecosystem-based adaptation’’ relies on natural or biological systems to mitigate natural disaster risks and to safeguard essential ecosystem service [35].

According to [7], the transformative agenda of IWRM is to assess the nature and status of the water resource, define short-term and long-term goals for the system, determine objectives and actions needed to achieve selected goals, assess both benefits and costs of each action, implement desired actions, evaluate the effects of actions and progress towards goals, and re-evaluate goals and objectives as part of an iterative process [18]. In essence, IWRM is supposed to be a dynamic and innovative planning, implementation support and Monitoring and Evaluation tool in the management of water resources.

### 2.2. Polycentric Governance

Governance refers to a whole range of institutions and relationships involved in the process of governing [36]. This includes both formal institutions, such as laws, official policies, and organizational structures, and informal institutions: the power relations and practices that have developed and the rules that are followed in practice. All water-management systems are polycentric to varying degrees [37]. Though river basins and watersheds are thought to be appropriate for water management, they encompass different political entities, an aspect that presents greatest difficult in terms of negotiation and intergovernmental (agency) agreements [7]. However, the setback is compensated for by the similarities of policy sectors, organisational levels, universality of management functions and integration requirements across countries alongside. Hence water is a natural candidate for linkage approaches because of its multiple uses in a broad range of policy areas.

Water is classified as a common pool resource. Literature suggests that, for the management of larger common-pool resources “nested” institutions are necessary [38]. These exist, for instance, in some large-scale irrigation systems, where local user groups are often responsible for the management of the smallest “tertiary” irrigation and drainage canals, and an association of user groups or government is responsible for the larger-scale infrastructure [39]. Attention to structural variables in existing institutional arrangements is critical, as such structures have potential for both negative and positive influences on participation at lower levels of implementation [40].

Polycentric institutional arrangements normally adopt adaptive governance approaches that are generally nested quasi-autonomous decision-making units operating at multiple scales [41]. Though polycentric governance systems are prone to challenges, the interaction and diversity across organizational levels may increase the diversity of response options in addressing uncertainty and change [42]. Adaptive governance systems often self-organize as social networks with team and actor groups that draw on various knowledge systems and experiences for the development of a common understanding and policies [43]. In the process, leadership, trust and vision emerge to transform management organizations toward a learning environment.

Though private or collective-action institutions have advantages at smaller spatial scales, state institutions are better suited for larger spatial scales because of their greater ability to coordinate across greater areas and larger numbers of users [42]. The possibility of potential coordination problems, higher transaction costs, and problems of democratic legitimacy in polycentric governance systems is suggested by [44]. On the basis of a case study in Rhode Island, fragmentation or duplication of authority was found to be as effective as centralised coordination mechanisms with potential for value addition on collaborative effort through reduction of transaction costs over time as the parties get to know each other better and manage to cooperate in relatively modest projects [45]. In essence collaborative and participatory approaches contribute new sources of knowledge and help align nexus assessments with stakeholders’ needs.

## 3. Ecological Public Health and Participatory Assessment and Planning Dilemmas

Participation is a central tenet in development planning discourses and refers to the inclusion of those who are affected or who can affect a decision [46]. The rationale for participation in natural resources management is largely informed by local resistance to state control of natural resources and need to address tragedy of the commons arguments [27]. Several authors [47,48], have reviewed broad spectrum of forms of public participation. Likewise, several authors have reviewed the advantages and limitations of participatory approaches. According to some of the findings [49,50,51], the authors concur that participation improves the quality of decision-making process and improves use of available information and creativity in the society. Moreover, participation improves public understanding of the management issues at stake, enhances transparency in decision making and might stimulate better coordination, monitoring and evaluation among government agencies. Public participation is particularly important where there are information, finance and power constraints in managing an issue effectively, as is usually the case in water management [49].

Alternative avenues, such as litigation, may emerge in situations where stakeholders are not fully involved in framing, analysing, generating, and implementing solutions to complex public problems [52]. This hampers progress towards achievement of project objectives. However, where stakeholders participate, the benefits to planning process are evident. In participatory modelling [53], concludes that participation increases better understanding, dialogue, out of thinking discipline, nuanced focus on relevant problems that confront the real drivers of change, and formulation of strategy and policy. In a systematic description and analysis of participatory processes in ten European river basins, stakeholders obtained a better understanding of the management issues at stake and got to know and appreciate each other’s’ perspectives. This opened up possibilities for win-win solutions, and tangible improvements for the stakeholders and for the environment [52].

In the Dee Basin (Scotland), for instance, contestation between the local community and the local authority on compliance with the Urban Wastewater Treatment compliance directive led to involvement of the local community and subsequent development of solutions that the authorities had not previously considered, such as the inclusion of wetlands in the original plan. As a result of community involvement, the initial proposal was changed, enabling an increase in amenity values, water quality, and biodiversity within the area and greater ownership of the solutions developed. In an analysis of public participation in water reuse projects in Georgia, Texas, and California, USA, substantively better decisions emerged when diverse interests, knowledge, and expertise were involved in decision making processes [52].

The devolution of natural resource management responsibility from the state to “communities” or local user groups has become a widespread trend that cuts across countries and resource sectors. However, devolution of control over resources from the state to local organisations does not necessarily lead to greater participation and empowerment of all stakeholders. In the ladder of participation [48], extensively discusses handicaps in participation discourses. The findings are particularly critical in the context of IWRM where multisectoral teams and elected community membership take stewardship of water projects and programmes. Participation is often undermined by power holders who have the ultimate power to judge the legitimacy and feasibility of advice. Secondly, the choice of members to the board or committees may be done with the aim of placating and advancing the agenda of the power elite. Such committees are likely to rubber stamp decisions and are vulnerable to power holders’ public relations and diversionary tactics. Furthermore, power elites may use technical jargon in intimidating the localites. This is worse in situations where community organisations lack the technical capacity to articulate their priorities [48].

One-way communication, a form of passive participation, is one of the superficial tactics power holders use to claim occurrence of community participation in rubberstamping decisions during planning processes. In such situations the community has less opportunity to influence the programme [46]. The shortcomings are reflected in [50,51] who identified lack of moderation, suspicion and window dressing as some of the shortcomings during conventional participatory processes. One of the pervasive cross cutting shortcoming in the participatory models has to do with intracommunity power relations and dynamics [38]. This could be true with respect to gender and culture of the facilitators and default/deliberate choice of one-way communication in consultative meetings in entrenching ‘passivity’.

Given that different actors with different agendas often subjectively interpret participation, there is need to account for interests and knowledges of all stakeholders and livelihood perspectives from political ecology lenses [27]. A “transformative” participation that empowers and transforms involved communities is proposed by [54], while, [55], conceptualizes participation from a two-way flow of communication perspective in terms of information dissemination to passive participants or “communication” and gathering information from participants or “consultation”. In this study, exploration for the integration of transformation angle by [54], and [55], concepts from communication for development lenses were carried out.

Communication for development is built around the principle of empowerment in the identification of problems, development of solutions and implementation strategies, monitoring and evaluation [32]. Empowerment refers to the capacity of people to make effective choices, envision alternatives, participate in decision making, negotiate with influence, control and hold accountable institutions that affect their lives and livelihoods [56]. Implicitly, an effective empowerment programme has to take place across the entire planning continuum of a project. Empowerment is central to community-based disaster risk reduction and resilience planning initiatives [57]. This is especially critical in situations that involve use of integrative models. The paradigm shift to empowerment has, as such seen an increase, in support for participatory planning approaches. Public participation is rooted in the concept of community development planning approaches that seek to improve the social and economic conditions of a community through voluntary cooperation and self-help effort [58]. Participatory planning resonates well with the principles underpinning community-based disaster risk reduction in public health.

The three basic components of communication for development are advocacy, social mobilisation and behavioural change (or behavioural development) communication. Effective communication relies on the synergistic use of the three strategic components [32]. Advocacy informs and motivates community leadership to create a supportive environment to achieve programme objectives and the related development goals. In essence, the advocacy component is aimed at changing policies, allocating resources, public dialogue and conversation on critical issues. Social mobilization engages and supports participation of institutions, community networks, social/civic and religious groups to raise demand for or sustain progress towards a development objective by strengthening participation in activities at the grass-roots level. The backbone of developing the social mobilisation component of a communication strategy comes from a combination of data, participant and behavioural analyses [32,33], as well as, Community input. Methodologies and approaches to stakeholder, participant and behaviour analysis and the end product in terms of a communication objective and strategy are provided by [32].

Behaviour change communication involves face-to-face dialogue with individuals or groups to inform, motivate, problem-solve or plan, with the objective to promote and sustain change. In agricultural extension and adoption of technologies, sustaining the change implies use of networks, attaining a critical mass of adopters and re-engineering of a given state to achieve a desirable action [59]. In this study, this is contexualised as ecological sustainability in terms of human action and package of technology that mediates positive public health outcomes. Implicitly, individual and collective action (agency) and participatory action is critical in achieving the outcomes at community level. This study argues that community livelihoods should be the basis of participatory planning interventions if public health risks are to be identified and mitigated.

Communication for development is the planned use of strategies and processes of communication in achieving development and behaviour change [60]. Communication for development is concerned with re-engineering diffusion to a process of innovation. Identifying the problem, stakeholder mapping, stakeholder engagement, critical evaluations and reviews, as well as, addressing the social costs in a participatory manner are some of the prerequisite in re-engineering the process [61]. Since human health is a product of human-environmental interactions [24], communication for development is a critical tool for transforming participatory processes and increasing community resilience. In the next section, review of the legal and institutional framework on water in South Africa in the context of ecological public health and community participation were done.

## 4. IWRM in the Context of South Africa’s Community Participation in Development and Management of Water Resources

South Africa is one of the countries classified as water scarce [22]. The low and variable levels of rainfall, high evaporation rates and hot climate have significant implications on water balance, access, utilisation and public health. Many communities in South Africa still rely on untreated or insufficiently treated water from surface resources such as rivers for their daily supply. The impact is reflected in a dramatic increase in the episodes of waterborne diseases since 2000 [21]. The high water pollution burden exacerbates the precarious situation and public health risks. Of the 223 river ecosystem types, 60% are threatened, with 25% of these critically endangered [20]. Less than 15% of river ecosystems are located within protected areas and are threatened and degraded by upstream human activities. Of the 792 wetland ecosystems, 65% have been identified as threatened and 48% as critically endangered. In response the government has developed a policy framework whose overall objective seeks to sustainably manage the water resources.

Meeting water conservation and water demand management targets, reforms to redress past racial and gender imbalances in access to water for productive uses while addressing poverty and inequality, establishment and decentralisation of water resources management institutions, regulation of water resources and water quality, as well as, the integration of monitoring and information management systems are among the major challenges in the implementation of the country’s vision for sustainable and equitable access to water [20]. Chapter 9 of the National water strategy revitalisation strategy 2 (NWRS2) recognises that water management operates within a social, economic and ecological environment, and for effective and integrated management of water resources, top-down consultation should be replaced by citizens’ participation [20]. Increased stakeholder participation is thus envisaged through community forums and civil society organisation structures with the aim of balancing decision making processes within a developmental water management agenda. Accordingly, the establishment of catchment management forums in strengthening participation of communities and other stakeholders within a catchment are envisaged [20].

Several legislations and institutions are involved with water management in South Africa. The legislations and institutions include the National Water Policy [62], the National Water Act (Act 36 of 1998) and the Water Services [63]. The Water Services Act delegates water quality monitoring and management to Water Services Authorities (WSAs), Department of Water Affairs (DWA), Department of Health (DoH), Local Government and Civil society [64]. The primary responsibility for ensuring the provision of safe drinking water and monitoring quality rests with WSAs. The traditional leaders (chiefs and headmen) are the heads of their communities and give guidance on many issues including development as given under Traditional Leadership and Governance Framework Act, 2003 [65]. Communities hold meetings at the traditional leaders’ courts where various issues including development are discussed. The traditional leaders’ courts could as such provide a forum for deliberation on local issues before they cascade to higher levels [66].

The National Water Act, aims at ensuring that the nation’s water resources are protected, used, developed, conserved, managed, and controlled in ways that take into consideration such factors as, inter alia, meeting the basic human needs of present and future generations, promoting equitable access to water, redressing past discrimination, facilitating social and economic development, and protecting aquatic and associated ecosystems. The need for equity and fair procedures is found throughout the Principles 12–25 of the water policy [62]. The National Water Act of 1998 divides South Africa into Water Management Areas and prescribes processes by which strategies and management institutions ought to evolve in Water Management Areas. It advocates the use of stakeholder participation principle in development of Institutional and management systems, Catchment Management Agencies, Catchment Management Strategies, and Water Users’ Associations.

The water policy in South Africa mirrors the policy sectors under the IWRM paradigm. The policy is anchored on post-apartheid constitutional order, international customs and practice and land mark international instruments such as the Mar del Plata (1997), the 1992 Dublin world conference on water and Environment, Agenda 21, the 1996 Stockholm Global Water Partnership (IWRM), ratification and domestication of international treaties and protocols such as the Ramsar on wetlands and shared water systems under SADC protocols [62]. The policy mirrors global trends on sustainability, water availability per person focus, concerns on rising pressure on water resources and need to address potential conflicts.

The reconstruction pillar, recognition of water as a common pool resource, and the objective of achieving equitable and sustainable utilisation is by default premised on management of externalities and disaster risk reduction pillar within the nexus and adaptation-sustainable development (ASMD) frameworks. The disaster management pillar is particularly relevant to ecological public health philosophy. The policy recognises and seeks to tackle the risks of existing weak solid waste and effluent on water resources through an improved Monitoring and Evaluation frameworks and enhancement of communication as decision support tool in the management, development and allocation of water resources. Ecological public health perspectives are by default inherent in the reconstruction pillar. In the next section we review some findings on ecological health perspectives and public participation in one of the water management area, Limpopo Province.

### 4.1. Unbundling Participatory Dilemmas from Ecological Public Health Perspective: The Case of a South Africa’s Water Management Area

**Figure 1:**
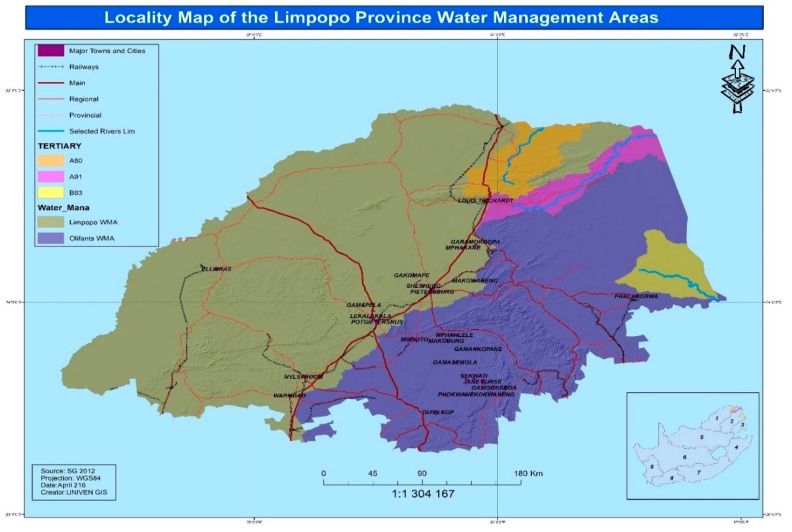
Locale of the Water Management Area studied (Source, GIS Generated, 2018).

The legal and policy framework in South Africa delegates the responsibility of monitoring domestic water quality to designated Municipalities. This part of the research focuses on findings generated by several authors about community participation on water quality monitoring from 3 Municipalities under Luvuvhu catchment and along River Nzhelele and the associated public health disaster risks. The review further considers findings on women participation and access to information on ground water exploitation from Mhinga and Lambani villages. Luvuvhu catchment consists of Vhembe, Makhado and Thulamela District municipalities. The locations of Rivers Nzhelele and Luvuvhu, on which the review is based are shown in Figure 1. River Nzhelele catchment covers an area of 2436 km^2^. The tributaries for River Nzhelele are Mutamba, Tshiruru, Mufungudi, Mutshedzi and Wyllie [67]. River Nzhelele is a major tributary of Limpopo River, a major water course in South Africa on coordinates 22°22′08″ S and 30°22′19″ E. It is a major alternative source of drinking water to villages along its course in Limpopo province of South Africa. The review is mainly based on some research findings on community participation in the Water management area by [66,68,69], and physicochemical analysis of drinking water from various sources by [70,71,72].

**Figure 2:**
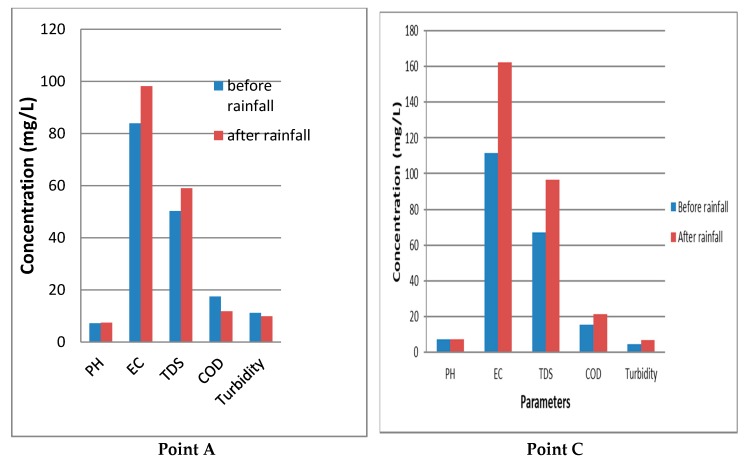
Physicochemical parameter differences between upstream and downstream sampling points on River Luvuvhu (Source, Authors Analysis of field data, 2018).

**Figure 3:**
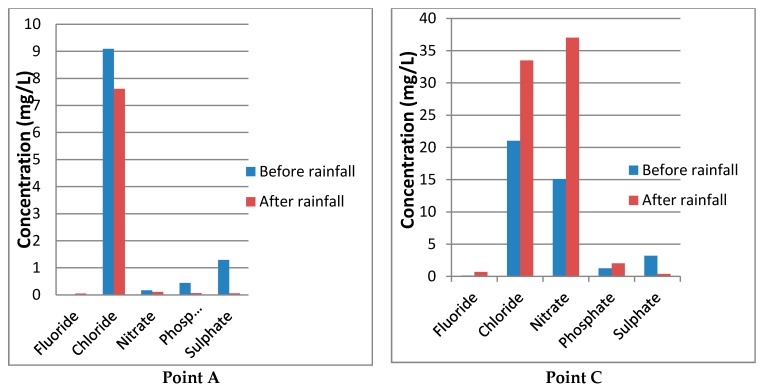
Chemical parameter differences between upstream and downstream sampling points on River Luvuvhu (Source: Authors Analysis, 2018).

To capture the Human-Environment interactions and potential impacts on public health, water was sampled from points 3 sampling points on River Luvuvhu, namely, upstream (point A), mid-stream (point B) and downstream (point C) before and after rainfall. The physicochemical parameters were analysed in the laboratory and compared with DWAF guidelines on water for domestic use [68]. The location of sampling Point C, captures cumulative human-environment interactions in terms of sources of pollution (agricultural activities, urban runoff and municipal waste water treatment plant). Point B is located near Levubu, a major agricultural area. Point A is majorly a conservation area within Louis Trichardt (near a hill and has a forest plantation) with isolated agricultural activities. The results of physicochemical analysis are presented in Figure 2 and Figure 3.

All natural waters contain varying concentrations of TDS as a consequence of the dissolution of minerals in rocks, soils and decomposing plant materials [73]. Table 1 provides levels of physicochemical parameters while Figure 2 provides a graph of the same. For sampling point A, the total dissolved solids (TDS) were 50.30 mg/L and 59.00 mg/L before and after rainfall respectively and within DWAF guidelines for domestic use. The chemical oxygen demand (COD) was 17.50 mg/L and 11.83 mg/L before and after rainfall and within the required range of 0–75 mg/L. However, turbidity levels of 9.87 NTU and 11.17 NTU before and after rainfall respectively were above recommended level.

According to DWA guidelines for domestic use, turbidity levels above 5 NTUs is visible and objectionable aesthetically undesirable to majority of water users. The high adsorption capacity of the particulate particles carries increased risk of microbial disease transmission, as well as, increase chemical toxicity.

Table 2 provides levels of chemical concentration at point A, B and C before and after rainfall while Figure 3 is a graph of chemical parameters. The electroconductivity (EC) at point B was 111.65 µS/cm before rainfall and within DWA guidelines for domestic use but increased to 162.43 µS/cm after rainfall which is above DWAF guidelines for domestic use. High EC at this point may be due to high ion content leached from agricultural fields. At Point C, mean EC was 139.87 µS/cm and 189.00 µS/cm before and after rainfall, respectively, and turbidity was 4.02 NTU and 9.93 NTU before and after rainfall, respectively.

The fluoride concentration at A are 0.003 mg/L and 0.05 mg/L before and after rainfall respectively and within the required range of (0–1.00 mg/L) as stipulated by DWAF [73] guidelines for domestic water use. Nitrate is the end product of the oxidation of ammonia or nitrite. Presence of nitrates in drinking water is a health concern because it is easily reduced into nitrite by gastrointestinal bacterial [74].

The health risks of nitrate are reviewed by [74]. The health risks associated with nitrate from food and drinking water are assessed through toxicological studies in animal studies [74,75], assessment of water supplies and nitrate exposure to trace cohorts for cancer incidences [75]. Toxicological studies have shown that presence of nitrosamine precursors increases the risk of cancer from consumption of nitrates. The potential effects of inorganic nitrate/nitrite on global health include methemoglobinemia and carcinogenic properties and anti-thyroid risks [74,75,76], with most cases of methemoglobinemia risks being reported in children [74]. Increased risk of thyroid cancer in women who consumed water from public water supplies with nitrates levels above 5 mg/l for more than five years has been reported [75]. However, no effect on thyroidal uptake and thyroid hormone disoders was observed among humans exposed to 15 mg/Kg intake of nitrates for less than one month [77]. This suggests that the health risks from consumption of nitrates are correlated with the length of time one is exposed to.

Nitrate concentration at A are 0.17 mg/L and 0.10 mg/L before and after rainfall respectively (Figure 3) and within the required range of (0–100 mg/L). However, the concentration at point C are 15.10 mg/L and 37.02 mg/L before and after rainfall respectively and above recommended range. The above results reveal some links between livelihood activities and public health risks. The social costs from such risks could be felt or incurred by members of the society who are not their cause or source. Mitigating such risks and externalities require individual, as well as collective action.

Lack of room for effective community input on water quality monitoring through formal policy and legislative frameworks is of great concern for community resilience and suggest the need for innovative alternatives. The observation that “Although the municipalities, as WSAs, are linked to the ward committees through the ward councillor and the Integrated Development plan (IDP), the linkages centre on other development issues other than water quality monitoring and management, a task that is handled by professionals in other departments and DOH excluding local participation”. For example, ”the Vhembe IDP document for 2008/2009 does not show any grants that are meant to support community-based monitoring and management”. Implicitly, implementation of IDP processes do not offer any opportunities for localised community-based water quality monitoring as the councillors do not consult communities before making presentation on the IDP [66]. In view of devolution, risk reduction gaps and participatory social learning frameworks, as well as, the need for sustainable and equitable outcomes envisaged under IWRM and the water Act, there is need for ecological public health perspective from social relations lenses.

Attention to structural variables in existing institutional arrangements is critical, as such structures have potential for both negative and positive influences on participation at lower levels of implementation [39]. Power influences social relations and right to access and manage natural resources [78,79]. One of the important institutions in the participation matrix in South Africa at the grassroot level is the ward committees. Ward committees are made up of elected representatives and are chaired by a councillor. Through the councillor, the ward committees have the power to make recommendations on any development matter to the metropolitan or local council. The structure is desirable as it is in sync with bottom up approaches that have potential to give the community a voice in matters that affect them.

Several researchers [70,71,72], have assessed microbial and physiochemical characteristics of water quality Nzhelele and Luvuvhu catchments. In an assessment of water quality parameters along Nzhelele River [71], researchers found that the levels of the major anions accessed were within the permissible limits of drinking water quality standards but there was high contamination from faecal organisms. This rendered the water unfit for human consumption. This suggests that though legal instruments in water quality monitoring may be effective in management of physiochemical aspects of water quality, they fail to address livelihood lenses (as an aspect of human health-environment interactions) and resulting biological public health risks at local levels.

Though the observation that development of cheap but efficient point of use water treatment devices could address the risk of water borne diseases [71], the participation from a gendered perspective is worth exploring. In a study of women participation in Limpopo province [69], provided evidence about gender-biased participation in community project planning frameworks and water projects in particular. He attributes the low participation by women to culture and low education among the women. In turn low levels of education among women and culture was found to influence access to quality water services. 

Inadequately treated water is embedded with health risks and impacts that can be linked to lack of policy and management. Reversing the undesirable conditions require adequate surveillance and integrated management from related fields. Integrated approaches are thus critical in understanding the state and causes of pollution and development of long term policies that improve water quality [80]. As part of an integrated approach to sustainable management of ground water resources in South Africa, Groundwater Resource Information Project (GRIP) was introduced, particularly in Limpopo province. The strategic objective of GRIP was about availing information to the community, planners and engineers [81]. However, in a study of Mhinga and Lambani villages of Nzhelele, lack of information about the basic processes on ground water among the community and implementation agencies on siting and licencing of new boreholes was more evident. The consequence was uncoordinated licencing of boreholes in excess of the available ground water capacity and the drying of most of the boreholes during critical times of the year [68]. The low yield of water from the boreholes impacted more on women as they are culturally bound to fetch water for the family. This suggests that participatory intent has failed to achieve its objectives. In view of public health risks, the effectiveness of community participation strategies envisaged under South Africa’s National Water Act of 1998 and IWRM, need to be reviewed by reviewing participatory frameworks for resilience building and disaster risk reduction with respect to water related public health risks.

### 4.2. Potential Solution to Participation Dilemmas in Water Quality Management

The policy and legal framework underpinning participation in water resources management in South Africa largely borrows from IWRM paradigm. Based on the literature review from the preceding sections core weakness of the paradigm are summarised in (Table 3) and use it to suggest an improved framework within the ecological public health perspective. Further suggestions of some of conceptual and analytical frameworks that can be pursued in the transformative agenda that integrates public health perspectives and empowers communities to take charge of water planning processes at large are made.

Responding to crisis, shaping change and building resilience for reorganisation and renewal of social-ecological systems involves linking a broad range of actors at multiple scales [25]. Water resources planning should take into account risk attitudes in policy making processes, seek to balance conflicting uses and mitigate adverse impacts from pollution [83]. Risk management provides the transition between problem identification (the all-hazards assessment step) and the mitigation [84]. Risk analysis tools are a prerequisite for public health sector planning. Hazard-capacity-vulnerability assessment (HCVA) provides some of the solutions. HVCA is a process used to identify strengths and weaknesses of households, communities, institutions and nations. It is an important tool that support decisions made in Disaster Risk Reduction [82]. The process of understanding vulnerabilities and capacities offer options as to how to contribute to the empowerment of communities at risk [82,85]. Since vulnerabilities are created or are products of economic, social development or faulty development, strategic planning, coordination and cooperation between agencies within the community is necessary in resilience building, especially in addressing challenges that are associated with interventions that address universal access to freshwater, inclusive economic growth and sustenance of key environmental systems functionality.

Disaster risk reduction is underscored as an overarching pillar for attainment of SDGs such as the ones on water [3]. Accordingly, there is need for improved understanding of disaster risks in all its dimensions of exposure, vulnerability, hazard characteristics, strengthening of disaster risk governance for prevention, mitigation, preparedness, recovery and rehabilitation [58]. A multi-hazard and multisectoral approach is envisaged in fostering people centered collaborative partnerships, mechanisms and institutions for implementation of instruments relevant to building resilient socioeconomic and ecological systems [86].

The assessment of possible disaster events (disaster risks) is a very important issue when mitigating disasters. The design of a comprehensive model revolves around six main components: strategic planning, hazard assessment, risk management, disaster management actions (four fundamental phases of disaster management), monitoring and evaluation and environmental effects [84]. This resonates with the ecological public health perspective. The results of, and assessments derived from the comprehensive model can be utilised as an input for a dynamic evaluation in a monitoring and evaluation system. The evaluation of all measures, and feedback to the strategic planning module is recommended in addressing environmental issues, an important aspect in ecological health perspective. Pollution and contamination levels as disaster risks in water can be assessed through Grey water footprints. Grey water footprints measures the amount of water required to assimilate a polluting load produced from human activity [87]. This approach can be used in valuation of externalities of human activities on water resources and the cost of mitigation.

In an evaluation of quality of water and spatial distribution of diarrhoea cases, from River Khandanama and ground water source used by Tshikuwi community in South Africa by [72], the presence of *Vibrio*, *Salmonella*, and *Shigella* species and the detection of total coliforms, faecal coliform, and enterococci counts, exceeded the acceptable limits and increased the vulnerability of the communities to episodes of diarrhoea. This is reflected in the outbreak of diarrhoea cases in the area [72]. Tshikuwi is located in Makhado municipality, Vhembe district, Limpopo province of South Africa. The geographic grid of the study area (Figure 1) is located between 22°55′ S and 22°57′ S latitude and 29°54′ E and 29°59′ E longitude.

Multidisciplinary approaches are critical in assessing socio-ecological interactions and impacts in water resources management and protection of water resources [87]. The rationale could be as a result of lack of clear distinction between individual and collective agency in natural resource (such as water) management. Hence the transformative potentials of individual agency and wider systems pathways of change remains a major challenge in adaptation discourses [88]. This is attributed to complex social networks and relations in which people are embedded and which influence commitments and understanding of social and ecological risks. Integrated modelling of interactions within and between socioecological systems as a strategy and objective of tackling complexity and uncertainties in contemporary water resources and land use management have been suggested [11]. Conceptual Modeling increases the possibility of synergy in decision making and application in adaptive processes that makes it possible to incorporate stakeholder input at any point and feedback. Scenario Modeling can be used to resolve uncertainty in real world through incorporation of representative spectrum of plausible alternatives for the future, exploring alternatives, assessing potential risks and opportunities and identifying response strategies to both opportunities and risks hence improved decision making.

Formal scenario planning requires participation of all stakeholders in scenario definition, scenario construction, analysis assessment and risk management. Participation increases credibility and legitimacy of information being produced hence effective decision making. However, integrated modelling on coupled human-environment systems is not yet mature enough for decision support and cannot provide adequate representations of the dynamics concerning the complex interactions between human and environmental components [11]. Although such models involve the stakeholders and decision-makers in the entire process of model development, implementation, and analysis with potential to enhance transparency and credibility of the modeling results, there are risks of decision-makers not selecting a scenario due to political or other concerns/considerations.

**Figure 4:**
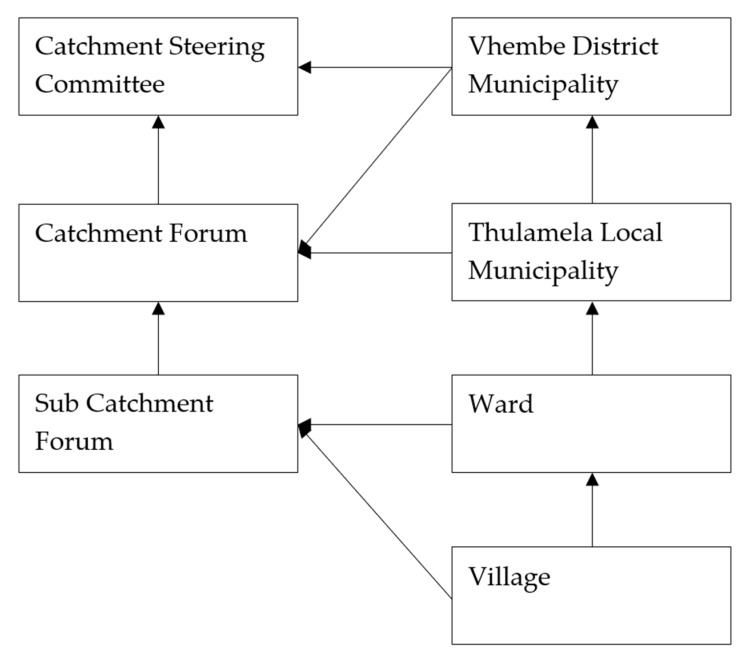
Proposed links between catchment management and local government structures (Source: Nare et al., 2011).

In assessment of community participation on water quality monitoring and management, in Luvuvhu catchment, Limpopo province of South Africa, the legal and policy framework seem to be supportive of community participation, but the integration of attitudes, indigenous knowledge, practices and leadership structures in the sustainable management of water resources though critical, remains low to non-existent [66]. The latter study proposes that the apparent policy-practice divide requires a community-based water quality management model anchored on a trialage of technology, empowerment and communication. Figure 4 gives the recommended model. The model recommends increased linkages between local government level structures and community level structures in improving the articulation of the opinions of the majority of the people and allowing information flow from the grass root level to the national level.

According to [66], 51% of sampled respondents could not identify with issues on water quality and only 5% of the community members were involved in monitoring of water quality in Luvuvhu catchment. More importantly, the existing information system did not integrate community perceptions on water quality and their monitoring. One of the reasons for the scenario is lack of representation by the community in the water quality monitoring committees. The observations resonate with other findings which reveal low community participation on use of available information on water resource management and planning e.g., [68]. The findings present a bleak scenario for public health related risks associated with water from various sources especially in the context of findings by [70,71,72].

A key theoretical argument through the case study on participation on water quality management and public health risks in Limpopo province is that participation could be better understood through political ecology and communication for Development lenses. Planning of water projects (especially identification and construction phases) is by default based on top-down planning models with the community being incorporated only at late stages e.g., through the establishment of water users associations as discussed in the previous sections. This planning approach negates policy interventions such as those envisaged under various acts of parliament in South Africa. The suggested model by [66], which is a bottom-up approach, has some potential in addressing the oversight. However, the reliance on structural lenses is prone to limitations cited by [39,40]. The gender aspect is particularly important in light of findings by [68]. We contextualise the weaknesses as identified in Table 3 to suggest a model that can be used to realise effective community participation in water quality monitoring. The model identifies community knowledge, attitudes and behaviour as critical elements that influence water quality outcomes. Our observation is particularly relevant in the context of observation by [72], that reducing health-risks from contaminated water supplies not only require the monitoring of groundwater and other water sources but also use of sustained health-education programmes in the communities. Thus, mitigating health risks associated with human-environmental interactions should focus on cognitive failures and livelihood lenses.

Strong environmental and conservation policies and legislation in many countries do not necessarily imply effective implementation and enforcement [89]. This observation is critical in our recommendation for a risk-based participatory community planning framework. In searching for resilient and sustainable water management system, advocacy, social mobilisation and behavioural change communication as integral part of communication for development should be used to influence community and other actors in the identification and planning of community water projects. A risk reduction-based planning model at community level has high potential to cure cognitive failures and mitigate water related public health risks. This resonates with ecological public health approaches.

**Figure 5:**
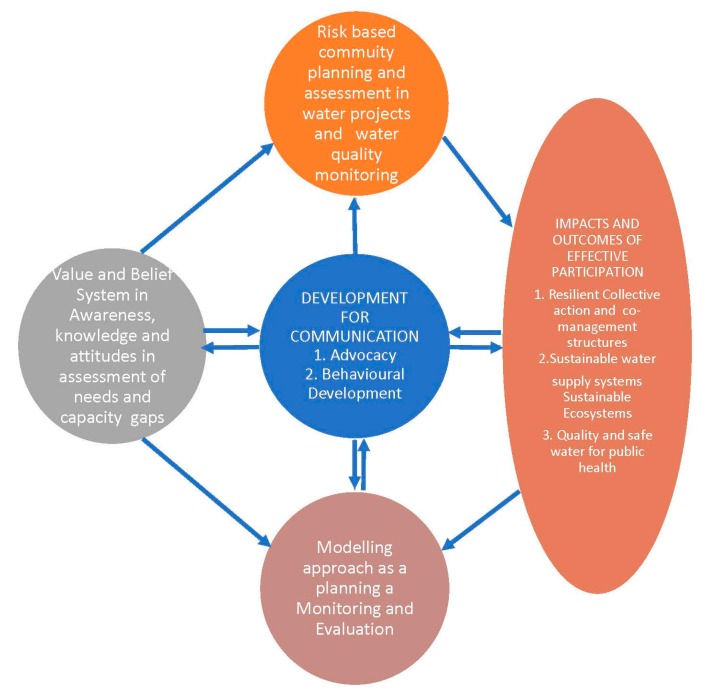
Community-based risk reduction model for interfacing planning and public health in water projects (Authors synthesis, 2018).

South Africa has one of the most vibrant legal and policy framework on water quality management. However, from the above literature, it is evident that though physiochemical risks are largely under control, microbiological risks are wide spread in rural community’s drinking water supply systems and a threat to public health. Available literature and our findings suggest such risks are livelihood related hence the need for a comprehensive risk-based community participatory planning framework on mitigation. The observation resonates with [71], in their recommendation for a comprehensive assessment of water quality and water use practices in rural areas of Limpopo. Given that all natural sources of water have presence of *E. coli* [70], recommended needs and risk assessments approaches in mitigation of water related health risks. Figure 5 presents the suggested community risk-based planning framework with communication for development lenses as the main pillar. 

## 5. Conclusions

In recent years, cooperative and collaborative efforts have become increasingly popular in ecosystem management, international and governmental policy. In response, policy makers and the community of practice are increasingly innovating and using integrative models in the analysis and resolution of challenges facing humanity. Over time, it has been realised that the inclusion of socioecological interactions and lenses in such models increases their utility, coherence and relevance as dependable tools for policy analysis and development planning. An ecological public health model is one of the models that fits this background. In recognition of the role water plays in public health and human welfare, the relevance of IWRM as a paradigm for transformation of water management globally through ecological public health lenses has been interrogated. The article has been contextualised to a water resource management area, Limpopo province of South Africa. We have extended political ecology lenses to ecological health in analysing community participation in water quality monitoring and its effect on public health risks. The focus on participation from institutional perspectives intended to enrich the utility and coherence of ecological public health model in integrating human agency from political ecology lenses. It is concluded that participatory planning, institutional and cultural change are some of the requisite dimensions in assessment of public health risks and building resilient social systems. This necessitates the assessment of knowledge, attitudes and existing practices and the extent to which they are integrated into water resources management and their integration with public health. Though the policy and legal frameworks recognise the role of participation, implementation of the same does not support integration of public health disaster risks. Communication for development has been realised to be central to unbundling cognitive failure and biases among planners, community, development practioners, and policy and decision makers. Since effective participation is dependent on utilisation of a two way communication feedback mechanisms to transform culturally embedded knowledge, attitude and behavioural norms (at individual, community, collective and institutional levels), the use of advocacy, behavioural change and social mobilisation is critical. In particular, a community-based planning approach for planning of water projects should encompass risk reduction principles in addressing health perspectives.

## Figures and Tables

**Table 1:** Physicochemical parameter differences between sampling points on River Luvuvhu.

Physiochemical Parameters	Point A		Point B		Point C	
	Before Rainfall	After Rainfall	Before Rainfall	After Rainfall	Before Rainfall	After Rainfall
Ph	7.20	7.41	7.33	7.12	7.46	7.24
EC (µS/cm)	83.90	98.17	111.65	162.43	139.87	189.00
TDS (mg/L)	50.30	59.00	67.02	96.57	83.93	101.93
COD (mg/L)	17.50	11.83	15.50	21.33	32.00	41.17
Turbidity (NTU)	9.87	11.17	4.53	6.62	4.02	9.93

Source, Authors Analysis of field data, 2018.

**Table 2:** Concentration of chemical parameters before and after rainfall.

Chemical Parameters	Point A		Point B		Point C	
	Before Rainfall	After Rainfall	Before Rainfall	After Rainfall	Before Rainfall	After Rainfall
Fluoride (mg/L)	0.003	0.05	0.21	0.18	0.14	0.70
Chloride (mg/L)	9.08	7.61	41.33	16.29	21.07	33.49
Nitrate (mg/L)	0.17	0.10	25.89	11.65	15.10	37.02
Phosphate (mg/L)	0.44	0.17	1.17	1.31	1.25	2.01
Sulphate (mg/L)	1.29	0.05	0.30	0.06	3.20	0.38

Source, Authors Analysis of field data, 2018.

**Table 3:** Identified weaknesses of IWRM model from ecological public health perspectives.

Pillar Dimensions	Weakness	Potential for Improvement	Possible Models for Improvement
Social	Less focus on, intra social relations, livelihood and human capital especially the local collective action in sustainability and risk reduction	Increased linkage between socioeconomic concerns and community-based multi risk assessment	Sustainable livelihood framework [49] Multihazard risk assessment (Carpignano et al., 2009)
	participatory assessment biased at policy and implementation agencies levels	focus on water-livelihood activities as part of Environment-human health matrix	ELS ((Biggs et al., 2015) Comprehensive models in Disaster risk Management (Asghar et al., 2006)
	Macro focus at expense of local access to water and livelihoods practices	Socio-ecological systems approaches and political ecology	Public health ecological perspectives (Lang and Raynor, 2012) Nexus Political ecology [23] ELS [82]
Economic	Focuses on allocative efficiency at the expense externalities	Livelihood and risk reduction	Comprehensive Disaster planning models (Asghar et al., 2006)
Environmental	Cognitive failure on management of externalities	Community-based planning and assessments	ELS (Biggs et al., 2015) Comprehensive Disaster planning models (Asghar et al., 2006)
Institutional	Centralised structural focus e.g., [13] Cognitive biases and failures		Comprehensive Disaster planning models (Asghar et al., 2006) Communication for development (e.g., UNICEF, 2008)
